# A Text Book of Normal Histology

**Published:** 1893-11

**Authors:** 


A Text-Book of Normal Histology, by Dr. George A. Piersol, Professor of Anatomy in the University of Pennsylvania, is announced by J. B. Lippincott Company, among other new medical publications, for early issue. The author is eminently qualified by learning, research, and experience as a teacher to prepare such an important work as this, and in the arrangement of the text shows a wise sympathy with the wants of the students. While his descriptions of the various tissues and organs are sufficiently full, he has carefully avoided such detail as would bewilder the learner. The numerous illustrations are excellent, and with few exceptions cover the entire field of normal histology.

